# Omp19 Enables *Brucella abortus* to Evade the Antimicrobial Activity From Host's Proteolytic Defense System

**DOI:** 10.3389/fimmu.2019.01436

**Published:** 2019-06-26

**Authors:** Karina A. Pasquevich, Marianela V. Carabajal, Francisco F. Guaimas, Laura Bruno, Mara S. Roset, Lorena M. Coria, Diego A. Rey Serrantes, Diego J. Comerci, Juliana Cassataro

**Affiliations:** Consejo Nacional de Investigaciones Científicas y Técnicas (UNSAM-CONICET), Instituto de Investigaciones Biotecnológicas Dr. Rodolfo A. Ugalde, Universidad Nacional de San Martín, Buenos Aires, Argentina

**Keywords:** bacterial protease inhibitor, Omp19, gastrointestinal route of infection, brucellosis, intracellular proteases

## Abstract

Pathogenic microorganisms confront several proteolytic events in the molecular interplay with their host, highlighting that proteolysis and its regulation play an important role during infection. Microbial inhibitors, along with their target endogenous/exogenous enzymes, may directly affect the host's defense mechanisms and promote infection. Omp19 is a *Brucella* spp. conserved lipoprotein anchored by the lipid portion in the *Brucella* outer membrane. Previous work demonstrated that purified unlipidated Omp19 (U-Omp19) has protease inhibitor activity against gastrointestinal and lysosomal proteases. In this work, we found that a *Brucella omp19* deletion mutant is highly attenuated in mice when infecting by the oral route. This attenuation can be explained by bacterial increased susceptibility to host proteases met by the bacteria during establishment of infection. Omp19 deletion mutant has a cell division defect when exposed to pancreatic proteases that is linked to cell-cycle arrest in G1-phase, Omp25 degradation on the cell envelope and CtrA accumulation. Moreover, Omp19 deletion mutant is more susceptible to killing by macrophage derived microsomes than wt strain. Preincubation with gastrointestinal proteases led to an increased susceptibility of Omp19 deletion mutant to macrophage intracellular killing. Thus, in this work, we describe for the first time a physiological function of *B. abortus* Omp19. This activity enables *Brucella* to better thrive in the harsh gastrointestinal tract, where protection from proteolytic degradation can be a matter of life or death, and afterwards invade the host and bypass intracellular proteases to establish the chronic infection.

## Introduction

The intestinal mucosa is the largest interface between the external environment and the tissues of the human body. The first line of defense in the gastrointestinal tract is in the lumen, where microorganisms are degraded in a non-specific fashion by pH and gastric, pancreatic and biliary secretions. Pathogenic microorganisms confront several proteolytic events in the molecular interplay with their host, therefore proteolysis and its regulation play an important role during infection. Microbes synthetize protease inhibitors to control endogenous proteases. Some inhibitors can also interact with exogenous peptidases produced by other species and thus may directly affect host's defense mechanisms (1). Few works in the literature show the importance of bacterial protease inhibitors activity against host-proteases (2–4). Our hypothesis is that pathogenic bacteria synthesize protease inhibitors to evade the antimicrobial activity from host's proteases.

In our laboratory, we have been working on the use of a conserved *Brucella* spp. protein devoid of its lipid moiety called U-Omp19 as a vaccine against brucellosis (5–7). Omp19 has significant sequence identity with bacterial protease inhibitors from I38 family. Remarkably, recombinant U-Omp19 inhibits gastrointestinal and lysosomal proteases (8, 9). However, the physiological function of Omp19 in *Brucella* is still unknown.

Brucellosis is a worldwide re-emerging zoonotic disease that is transmitted from domestic and wild animals to humans. The human disease, mostly caused by *Brucella abortus* and *B. melitensis*, represents an important cause of morbidity worldwide whereas animal brucellosis is associated with serious economic losses caused mainly by elicited abortions and infertility (10, 11).

Oral infection is one of the principal ways of brucellosis transmission. Animals usually lick tissues from abortions or ingest contaminated pasture and humans acquire often the disease by consumption of infected, unpasteurized dairy products (10, 12–16). Few virulence factors required for food-borne infection by *Brucella* have been described: Urease and cholylglycine hydrolase that confer resistance to gastric acidity and bile salts, respectively (17, 18). Once inside the host, *Brucella* disseminate via infected phagocytic cells to different tissues and organs, developing foci of infection, surviving intracellularly and leading to a chronic disease (19).

Digestive enzymes, primarily proteases, contribute to the non-specific host defense system exerting a toxic action on microorganisms by destruction of their cell wall (20). Omp19 is a lipoprotein anchored in the *Brucella* outer membrane (7). This location together with its protease inhibitor activity suggest that it may play a protective role against host proteases.

In this work, we studied if Omp19 enables *Brucella* to better thrive in the harsh gastrointestinal tract, where protection from proteolytic degradation can be a matter of life or death, and thus promoting host invasion and intracellular infection.

## Materials and Methods

### Ethics Statement

Protocols of this study agreed with international ethical standards for animal experimentation (Helsinki Declaration and amendments, Amsterdam Protocol of welfare and animal protection and NIH guidelines for the Care and Use of Laboratory Animals). Protocols of this study were approved by the Institutional Committee for the Care and Use of Experimentation Animals from UNSAM (CICUAE-UNSAM_N°04/2014).

### Bacterial Strains, Media, and Culture Conditions

*Brucella* strains were derived from the wild type (wt) 2308 biovar and were: (i) smooth virulent wt *B. abortus*; (ii) unmarked *omp19* deletion mutant (Δ*omp19*); and (iii) *omp19* complemented Δ*omp19* mutant (Δ*omp19*pBBR4*omp19*). All strains were grown as described in Czibener and Ugalde (21). When necessary, media were supplemented with the Ampicillin (100 μg/ml) or Nalidixic acid (5 μg/ml). CFU determination from intestine containing samples were performed in medium with following antibiotics to inhibit normal flora growth: Vancomycin (20 μg/ml), Cycloheximide (100 μg/ml), Bacitracin (10 U/ml), and Nalidixic acid. All work with live *Brucella* was performed in BSL3-laboratories and BSL3-animal facility at UNSAM. *Escherichia coli* strains were grown at 37°C in LB with Ampicillin.

### Generation of Mutant Strains

#### (i) Δomp19 Strain

Omp19 (BAB1_1930) unmarked chromosomal mutant was generated as described in Herrmann et al. (22). Briefly, two DNA fragments of ~500 bp containing flanking regions of BAB1_1930 were amplified from *B. abortus* 2308 genomic DNA. Primers used to amplify omp19′s upstream regions were: omp19(EcoRI)_Up_Fw_5′-GAATTCTCGAAGGCTGTTTCGCTATCG-3′ and omp19_Up_Rv_5′- CAGGTTCTCCATTTGCGCATTT-3′; and omp19_Down_Fw_5′-CAAATGGAGAACCTGTCTGACCCGGAAACGATGAAC-3′ and omp19(BamHI)_Down_Rv_5′-GGATCCTTGTGCGCCTGACGATGC-3′ for downstream region. Fragments were ligated by overlapping PCR using omp19(EcoRI)_Up_Fw and omp19(BamHI)_Down_Rv. The resulting fragment was digested with EcoRI and BamHI, cloned into pK18mobSacB (23) and conjugated to *B. abortus* 2308 by biparental mating. Single recombinants selection, selection with sucrose, excision of plasmids, and generation of deletion mutants was performed as described previously described (21). Deletion of BAB1_1930 was confirmed by PCR and sequence analysis and western blot (Figure S1).

#### (ii) Complementation of Δomp19 Mutant

A 1000 bp DNA fragment containing the complete gene (BAB1_1930) and its promotor was amplified using primers Omp19(BamHI)_ATG_5′-ATGGATCCATGGGAATTTCAAAAGCAAGTCTGC-3′ and Omp19(SpeI)_TGA_5′-GAACTAGTTCAGCGCGACAGCGTCA-3′, digested with BamHI and SpeI and ligated into pBBR4 to generate the plasmid pBBR4*omp19*. This plasmid was electroporated into Δ*omp19* mutant. The resulting complemented strain was called Δ*omp19*pBBR4*omp19*. Complementation was confirmed by PCR and western blot (not shown).

### Recombinant Proteins, Enzymes, and Extracts

Mouse intestine- and stomach-extracts were obtained as previously described (8). Briefly, intestines and stomachs extracts were obtained from 6 to 12 weeks old female or male Balb/c mice (*n* = 10). Prior to fluid preparation, mice were fasted for 2.5 h (water ad lib.) and euthanized by CO_2_ inhalation. Stomachs and small intestines were resected, homogenized in PBS, and fluid separated by centrifugation (10 min, 13,200 × g at 4°C). Pooled Intestinal or stomach fluids were snap-frozen in liquid nitrogen and stored at −80°C. Protein concentration and proteolytic activity were determined as previously described by Ibañez et al. (8). Microsomes of J774 murine macrophages were obtained as described previously by Coria et al. (9). Pancreatin, Elastase, and Trypsin from pig and α-Chymotrypsin from bovine were from Sigma.

Recombinant U-Omp19 was produced as previously described by Pasquevich et al. (5). For Omp25 production, the complete sequence of *B. abortus* omp25 gene (GenBank_X79284.1) (24) was synthetized and subcloned into pET22(b)+ (Novagen) in frame with 6 × His-tag (genscript). Expression and purification was performed as described in Goel and Bhatnagar (25).

### Infection of Mice

Six to eight-week-old female BALB/c mice were bred at IIB-UNSAM. Five mice/group were inoculated (i) wt, (ii) Δ*omp19*, or (iii) Δ*omp19*pBBR4*omp19 Brucella* strains either by gavage (i.g.) with 1 × 10^9^ CFU in 0.2 ml PBS (18, 26) or with 1 × 10^10^ CFU directly into the oral cavity as previously described by von Bargen et al. (27). Infected mice were kept in cages within a BSL3 facility. At different times post-infection mice were euthanized by CO_2_ inhalation and organs were aseptically collected, homogenized, and plated for CFU determination. Intestinal samples were plated on TSA supplemented with Vancomycin, Cycloheximide, Bacitracin, and Nalidixic acid. In some experiments tissue samples from duodenum were obtained for immunofluorescence analysis.

### Intestinal Tissue Immunofluorescence

Duodenum sections from mice infected i.g. either with wt or Δ*omp19 B. abortus* were excised, fixed (4% paraformaldehyde), immersed in 30%-sucrose buffer, embedded in OCT-medium and frozen (-80°C). Cryosections (10 μm) were mounted on positively charged glass-slides (Biogenex), permeabilized with 0.2% Tween20 and blocked with 1% BSA and 5% horse serum in PBS. *Brucella* detection was performed as previously described (21). RNAse A (10 μg/ml) treated samples were counterstained with Alexa-Fluor555-WGA (ThermoFisher) and TO-PRO^®^-3 (Invitrogen). Sections were mounted using FluorSave reagent (Calbiochem) and images obtained on an IX-81 Olympus microscope with FV-1000 confocal module. A ROI was set for each treatment, background subtracted and images merged (RGB) (ImageJ software, NIH).

### Bacterial Susceptibility to Proteases

#### (i) Agar Disk-Diffusion Method

*Brucella* strains (1 × 10^8^ CFU) were spread on TSA plates supplemented with Vancomycin, Cycloheximide, Bacitracin, and Nalidixic acid. Five-mm filter disks impregnated with either PBS, intestine- or stomach-extract were placed on the agar surface. After 72 h of incubation (37°C) zones of inhibition were determined.

#### (ii) Protease Broth-Susceptibility Test

*Brucella* strains (1 × 10^5^ CFU/ml) were incubated in 10% TSB plus buffer, intestine-extract (8.5 mg/ml), pancreatin (2 mg/ml), α-chymotrypsin (50 μM), trypsin (20 μM), pancreatic elastase (5 μM), or microsomes from J774 macrophages (2 mg/ml) for the different periods of time at 37°C. Negative control was buffer supplemented with 10% TSB. Buffer was PBS (intestine extract or microsomes), 0.5% ClNa (pancreatin), 10 mM Tris-HCl, pH7.8 (α-chymotrypsin and trypsin), or 10 mM Tris-HCl pH8.8 (pancreatic elastase). All protease solutions were sterilized by filtration before to incubation with the bacteria. Live bacteria (CFU/ml) were determined at different time points by serial dilutions plating.

### Bacterial Growth Analysis

*Brucella* strains were labeled with TRSE (Invitrogen) as previously described by Brown et al. (28). Bacteria were spotted on 1% agarose pads with 10% TSB plus PBS or pancreatin (2 mg/ml). Images were obtained before and after 24 h of culture on an Olympus IX-81 microscope with FV-1000 confocal module. Images were subtracted the background and merged using RGB format (ImageJ software). Number of total bacteria (N) and initial number of bacteria (N_0_, number of labeled or partially labeled bacteria) were enumerated using Spot Detector plugin (ICY software, Institute Pasteur). Three to nine images/condition in duplicates were evaluated (50–150 colonies/condition). Then, assuming exponential growth, the average number of cell divisions (n) was calculated:

$$Average\;number\;of\;cell\;divisions=n=\log_{2}\frac{N}{N_{0}}\;$$

### DNA Content on Individual Bacteria

*Brucella* in exponential phase (5 × 10^7^ CFU/ml) were incubated with or without pancreatin (2 mg/ml). After 1.5–6 h, cells were washed, fixed, incubated with RNase A and labeled with SYTOX-Green (Invitrogen). Samples were analyzed in a FACS ARIA II (BD Biosciences) and analyzed with FlowJo7.6.2 software (Tree Star).

### Western Blot

*Brucella* strains (5 × 10^8^ CFU/ml) were cultured with 10% TSB buffer with or without pancreatic elastase (10 μM), washed and boiled in sample buffer (5 min). CFU/ml were determined in a sample taken prior to stop the reaction and 1 × 10^7^ CFU/lane were subjected to SDS-PAGE and transferred onto nitrocellulose membranes. Immunoblotting was performed using mouse monoclonal antibodies against Omp1, Omp2b, Omp25, Omp10, Omp16, and Omp19 (29), rabbit polyclonal anti-CtrA (30) or mouse anti-GroEL serum, followed by incubation with anti-mouse-IgG-HRP (Sigma) or anti-mouse-IgG IRDye antibodies (Li-Cor Biosciences). Images were acquired with Odyssey image-scanner and band intensities (RFU) were quantified (Image-Studio-Lite Software). Omp16, Omp10, and GroEL were similar in all treatments and served as loading control. Percentage of digested Omp25 was calculated:

$$\begin{array}{l} percentage\;of\;digested\;Omp25\;=\frac{Digested\;Omp25\;RFU/lane}{\;Total\;Omp25\;RFU/lane}\times \;100. \end{array}$$

### Omp25 Digestion

Purified Omp25 (1 μM) was incubated with pancreatic elastase (1 μM) or buffer (10 mM Tris-HCl, pH8.8) with or without U-Omp19 (45 μM). Reactions were stopped by sample buffer addition and boiling. Omp25 digestion was followed by western blot.

### Cell Culture and Infection Assay

J774 macrophages were maintained in RPMI 1640 supplemented with 5% fetal bovine serum (FBS) and streptomycin (50 μg/ml)-penicillin (50 U/ml) (Gibco Life Technologies) in a humidify 5% CO_2_ atmosphere at 37°C. Cells (5 × 10^4^ per well) were seeded on 24-well plates in antibiotic-free medium and were kept for 24 h. *B. abortus* infections were carried out at a multiplicity of infection (MOI) of 500:1 or 100:1. After a 1 h incubation with the bacteria, wells were washed three times with PBS and incubated with fresh medium containing 50 μg/ml of Gentamycin and 100 μg/ml streptomycin to kill non-internalized bacteria. At the indicated time points, infected cells were washed three times with PBS and lysed with 500 μl 0.1% Triton X-100 (Sigma-Aldrich). The intracellular CFU were determined by plating serial dilutions on TSA. In some experiments, prior to the infection of cell the bacteria were incubated at 5 × 10^7^ CFU/ml with or without pancreatin (2 mg/ml) during 2 h. Afterwards bacteria were washed and suspended in medium to infect the cells.

### Statistical Analysis

Statistical analysis and plotting were performed using Prism^®^ 7.04 (GraphPad, Inc., USA). CFU data were logarithmically transformed. Unpaired two-tailed Student *t*-test was used for pairwise comparisons between means of two groups or one-way or two-way ANOVA followed by Bonferroni's posttest was used for comparing more than two means. Significance level was set at *p* < 0.05.

## Results

### Omp19 Expression Is Needed for Oral Acquired *B. abortus* Infection

To investigate the role of Omp19 in *Brucella* infection, a deletion mutant (Δ*omp19*) and its complemented strain (Δ*omp19*pBBR4*omp19*) were constructed in the *B. abortus* wild-type (wt) strain 2308.

Mutant and wt strains had similar growth curves, resistance to low pH and bile salts. Moreover, membrane permeability to hydrophobic substances, expression of main outer membrane proteins (Omps) (Omp1, Omp2b, Omp25, Omp10, and Omp16) and lipopolysaccharide O-antigen were similar between wt and Δ*omp19* strains (Figures S1A–F). The authenticity of the mutant was verified by PCR and immunoblot analysis on whole-cell extracts with an anti-Omp19 Mab (Figures S1E,F).

**Figure 1:**
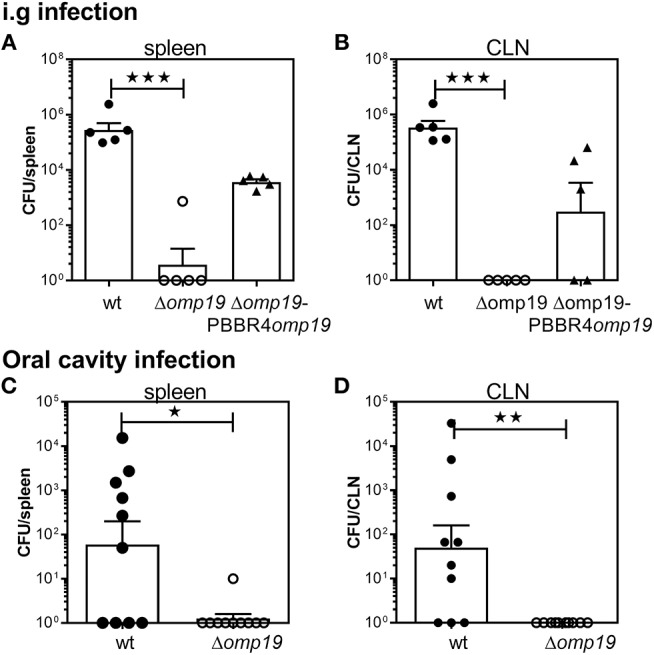
*B. abortus* Δ*omp19* is attenuated after oral infection. **(A,B)** BALB/c mice were i.g. inoculated with 1 × 10^9^ CFU/mouse of wt, Δ*omp19* or Δ*omp19*pBBR4*omp19 B. abortus* strains. Spleen and CLNs were homogenized and plated for CFU counting at 20 days post-infection (^***^p < 0.001 vs. wt *B. abortus*). Results are representative of three independent experiments with 5 mice per group. **(C,D)** BALB/c mice were inoculated at the oral cavity with wt or Δ*omp19 B. abortus* (1 × 10^10^ CFU/mouse). Twenty days after infection the number of CFU at the spleens and CLNs were quantified (^*^p < 0.05 and ^**^p < 0.01 vs. wt *B. abortus*). Pooled data from two independent experiments are shown. Each point represents data from an individual mouse. Horizontal lines and error bars represent the mean ± s.e.m. (For some mice, no live bacteria were recovered from spleens or CLNs. These were arbitrarily assigned with values of 1 CFU).

To evaluate the role of Omp19 in the establishment of *B. abortus* infection through the digestive tract *in vivo*, BALB/c mice were inoculated intragastrically (i.g.) with wt, Δ*omp19* or Δ*omp19*pBBR4*omp19* and 20 days post-infection bacterial loads at spleens and cervical lymph nodes (CLNs) were assessed. While wt and Δ*omp19*pBBR4*omp19* established infection, there were significant lower numbers of CFUs at spleens and CLNs from Δ*omp19* infected mice (*p* < 0.001 vs. wt) (Figures 1A,B).

Upon gavage administration, initial events of bacterial invasion and onset of infection in the oral cavity may be bypassed. Thus, BALB/c mice were administered directly into the oral cavity as described in von Bargen et al. (27) with wt or Δ*omp19*. Twenty days post-infection *B. abortus* were isolated from spleens and CLNs from wt infected mice, whereas almost no CFUs were found in these organs of Δ*omp19* infected mice (*p* < 0.05 and *p* < 0.01 vs. wt, respectively) (Figures 1C,D).

Altogether, these results demonstrate that Omp19 plays a crucial role in the establishment of infection by *Brucella* through the oral route in mice.

### *Brucella abortus* Reaches Intestinal Tissues Upon Oral Infection and Requires Omp19 to Evade the Bacteriostatic Action of Intestinal Content

**Figure 2:**
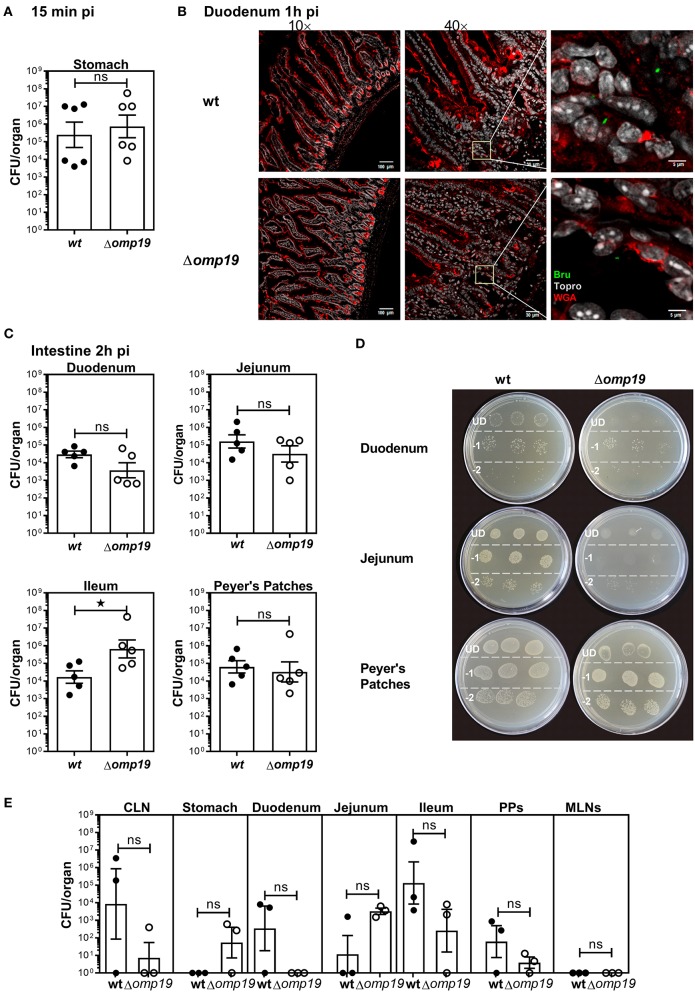
*Brucella abortus* requires Omp19 to evade the bacteriostatic action of intestinal content. BALB/c mice were i.g. inoculated with (1 × 10^9^ CFU) of wt or Δ*omp19 B. abortus* strains. **(A)** Total CFUs per stomach in animals sacrificed at 15 min post-infection. Each point represents an individual mouse, horizontal lines, and error bars represent the mean ± s.e.m. **(B)** Confocal microscopy images of duodenum of infected mice at 1 h post-infection. The images correspond to ROI merged signals for *Brucella* (green channel), mucin (WGA) (red channel) and nuclei (TO-PRO-3, NIR channel). The inset region of middle images (40×) was magnified and presented in the right, showing individual Brucellae in the epithelium. Scale bars are: 100 μm (left panels), 30 μm (middle panels) and 5 μm (right panels). **(C)** Total *B. abortus* CFUs recovered from duodenum, jejunum, Ileum or Peyer's Patches from infected mice sacrificed at 2 h post-infection. **(D)** Representative agar plates showing sequential 1:10 dilutions and drop plating from depicted tissues homogenates from wt or Δ*omp19* infected mice. UD (undiluted), −1: 1 to 10 dilution; −2: 1 to 100 dilution. Results are representative of two independent experiments. **(E)** BALB/c mice were inoculated at the oral cavity with wt or Δ*omp19 B. abortus* (1 × 10^10^ CFU/mouse). Two hours after infection the number of CFU at CLNs, Stomach, Duodenum, Jejunum, Ileum, PPs, and MLNs were quantified. E*ach* bar represents the mean CFU/organ (logarithmic sc±ale) and error bars represent the mean ± s.e.m. (For some mice, no live bacteria were recovered, these were arbitrarily assigned with values of 1 CFU). (Statistical analysis was performed by unpaired *t*-test to compare between the indicated groups: ^ns^*p* > 0.05; ^*^p < 0.05).

To evaluate if Δ*omp19* attenuation after oral infection is due to higher susceptibility to gastrointestinal content, short-term gavage infection experiments were performed. BALB/c mice were i.g. inoculated with wt or Δ*omp19* strains and at different time points post-infection the stomach and intestinal sections were analyzed. After 15 min equal numbers of bacteria were isolated from the stomachs of both groups (Figure 2A). After 1 h both strains were present in the duodenum at the lumen as well as in the epithelium (Figure 2B).

Next, *B. abortus* loads in different sections of the small intestine: duodenum, jejunum, ileum and Peyer's patches were evaluated. Almost no differences in wt and Δ*omp19* CFUs were detected at 2 h post-infection with a slight but significant increase in Δ*omp19* CFUs at Ileum (Figure 2C) that may not explain the attenuation of this strain when infecting by the oral route. However, when plated undiluted (direct plating from each tissue on TSA + Antibiotics) low-density bacterial growth and small colonies were found in the drops of Δ*omp19*, indicating that the intestine content impaired Δ*omp19* strain's growth. This effect was temporarily and reversible, since upon dilution it disappeared and both, wt and Δ*omp19*, showed similar numbers and phenotype of colonies (Figure 2D). These results indicate that the intestine content exerts a bacteriostatic action on Δ*omp19*, suggesting that Omp19 protects *Brucella* from intestinal proteases.

Similar results were obtained when bacteria where inoculated directly at the oral cavity of mice. Both strains, wt and Δ*omp19*, were recovered from intestinal tissues after 1 h of infection (Figure 2E), indicating that *Brucella* reaches the intestine after oral infection (by gavage or oral cavity delivery) and there it is exposed to the intestinal content that exerts a bacteriostatic effect.

### Omp19 Protects *B. abortus* Against the Action of Pancreatic Proteases

To further assess the role of Omp19 against the action of gastric and gut content, *in vitro* bacterial susceptibility assays were performed.

**Figure 3:**
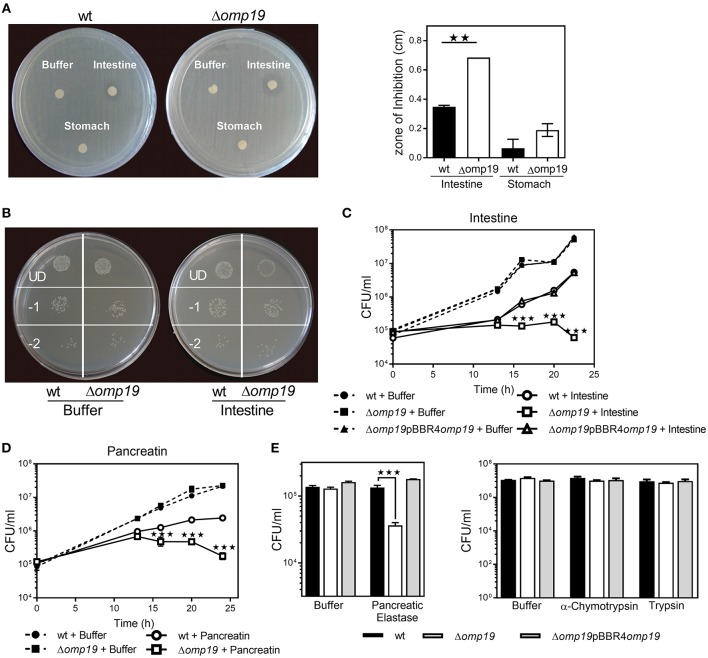
Omp19 protects *B. abortus* against the action of pancreatic proteases. **(A)** 1 × 10^8^ CFU of wt and Δ*omp19 B. abortus* were spread on TSA plates supplemented with antibiotics. Five-mm filter-disk were impregnated with either PBS, intestine- or stomach-extract and placed on the agar surface. The plates were incubated at 37°C for 72 h and afterwards the diameter of the zones of inhibition were determined (diameter of no growth zone minus diameter of the disk). (^**^p < 0.01 vs. *wt B. abortus* + intestine extract). **(B)** Representative picture of a plate with *wt* and Δ*omp19 B. abortus* treated with buffer or intestine extract. Plated undiluted (UD) or after serial dilutions: 1/10 (−1) and 1/100 (−2). **(C)** *wt*, Δ*omp19* or Δ*omp19*pBBR4*omp19 B. abortus* strains (1 × 10^5^ CFU/ml) were incubated with PBS or intestine extract at 37°C. Live bacteria (CFU/ml) were determined after 12, 16, 20, and 24 h of incubation by serial dilutions plating (^***^p < 0.001 vs. *wt B. abortus* + intestine extract). **(D)** *wt* and Δ*omp19 B. abortus* (1 × 10^5^ CFU/ml) were incubated with buffer (0.5% ClNa) or pancreatin (2 mg/ml). Live bacteria (CFU/ml) were determined after 12, 16, 20, and 24 h of incubation by plating serial dilutions on TSA (^***^p < 0.001 vs. *wt B. abortus* + pancreatin at the same time point). **(E)** wt and Δ*omp19 B. abortus* (1 × 10^5^ CFU/ml) were incubated with buffer (10 mM Tris-HCl, pH8.8) or pancreatic elastase for 5 h or with buffer (10 mM Tris-HCl, pH7.8), α-chymotrypsin or trypsin for 24 h. Live bacteria (CFU/ml) were determined by plating serial dilutions on TSA (^***^p < 0.001 vs. wt *B. abortus* + pancreatic elastase). Results are representative of two or three independent experiments.

An agar disk-diffusion test indicated that Δ*omp19* is more susceptible to the action of intestine content than the wt strain (*p* < 0.01 vs. wt + intestine extract) (Figure 3A), whereas stomach content did not affect bacterial growth.

Incubation with intestine-extract inhibited Δ*omp19*'s growth and this action was bacteriostatic, since viable bacteria were recovered by dilution (Figure 3B). Viable bacteria determination over time indicated that in presence of intestine-extract Δ*omp19* was unable to grow (*p* < 0.001 vs. *wt* + intestine), whereas the wt and the complemented strains grew exponentially after 13 h of culture (Figure 3C). Similar results were obtained using pancreatin (a pig pancreatic extract) (Figure 3D), supporting that *B. abortus* requires Omp19 to grow when exposed to intestinal content.

As purified U-Omp19 inhibits main gastrointestinal proteases (8), the effect of individual proteases (pancreatic elastase, α-chymotrypsin, trypsin) on wt, Δ*omp19* and Δ*omp19*pBBR4*omp19* viability was assessed. Δ*omp19* was more susceptible *in vitro* to pancreatic elastase action than wt and Δ*omp19*pBBR4*omp19* (*p* < 0.001). In contrast, α-chymotrypsin and trypsin did not alter bacterial growth (Figure 3E).

These results together demonstrate that *B. abortus* requires the expression of Omp19 to resist the action of intestinal proteases.

### Δ*omp*19 *B. abortus* Stops Cell Division and Cell-Cycle Progression at G1-Phase After Incubation With Pancreatic Proteases

**Figure 4:**
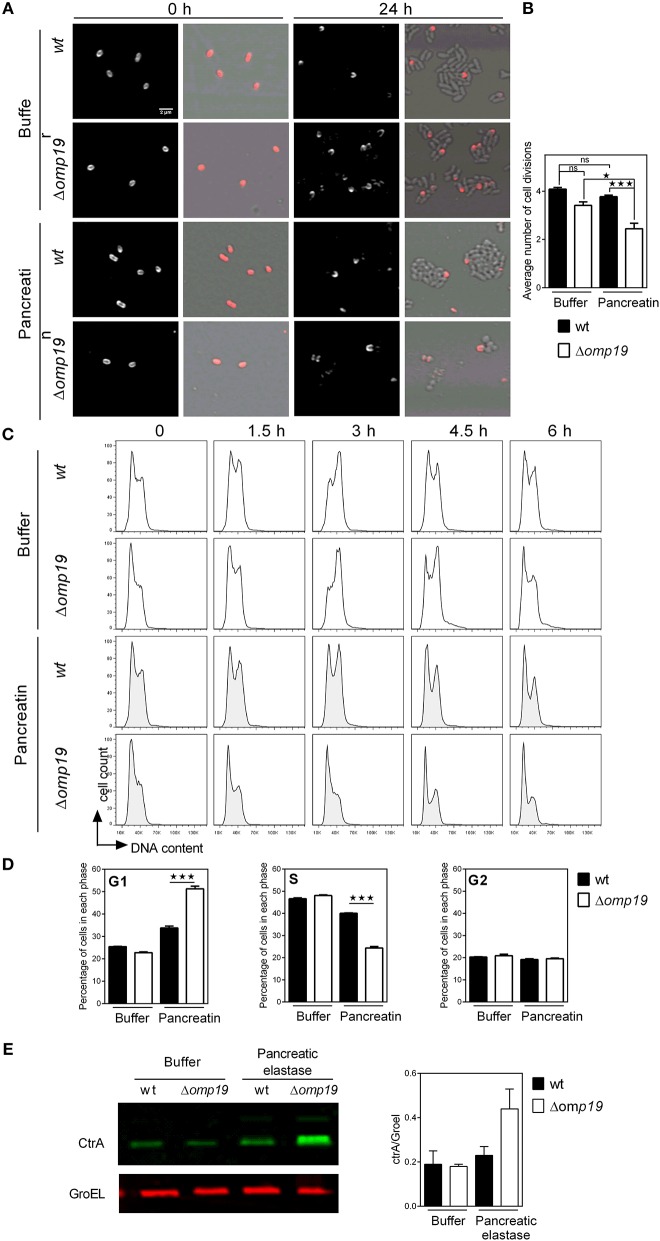
Δ*omp19 B. abortus* has a cell division defect and cell cycle arrest upon incubation with pancreatic proteases. TRSE-labeled *wt* and Δ*omp19 B. abortus* were dropped on TSB-agarose pads containing buffer or pancreatin and cultured for 24 h at 37°C. **(A)** Representative Texas Red fluorescence (left) and phase contrast microscopy images (right) from the beginning of incubation (0 h) and after 24 h of incubation are shown. **(B)** Average number of cell divisions after 24 h of culture obtained by quantification of the number of labeled (or partially labeled) cells and unlabeled cells in each individual colony (^*^p < 0.05 and ^***^p < 0.001 vs. *wt B. abortus* strain in pancreatin). Results are representative of two independent experiments. **(C)** Flow cytometry analysis of DNA content on individual bacteria. *wt* and Δ*omp19 B. abortus* were incubated in buffer or pancreatin for the indicated time periods and the content of DNA was evaluated by Flow cytometry. Representative histograms are shown. Results are representative of two independent experiments. **(D)** Bar graphs indicate the percentage of cells in each phase of cell cycle after 3 h of culture. (^***^p < 0.001 vs. *wt B. abortus* strain in pancreatin). **(E)** *B. abortus* wt and Δ*omp19* strains were incubated with buffer or pancreatic elastase. Equal quantities of bacteria were subjected to SDS-page followed by western blot analysis using specific antibodies for CtrA and GroEL. Images are representative of two independent experiments. The ratio of CtrA and GroEL signals was evaluated by quantitative analysis of western blot images. Bar graph represent the mean ± s.e.m. of pooled results from two independent experiments.

To evaluate if Δ*omp19*'s growth impairment when exposed to proteases is due to a cell division defect, *Brucella*'*s* growth was studied by microscopy. *Brucella* Texas-red succinimidyl-ester (TRSE) labeling allows, after growth, the visualization of an unlabeled pole and subsequently unlabeled or partially labeled daughter cells. TRSE-labeled *wt* and Δ*omp19* were cultured on TSB-agarose pads containing buffer or pancreatin. After 24 h wt and Δ*omp19* in buffer-pads and wt in pancreatin-pads formed microcolonies with many unlabeled cells surrounding partially labeled cells. However, Δ*omp19* in pancreatin-pads formed no or small colonies (small chains) with few unlabeled sphere-shaped bacteria (Figure 4A), indicating a cell division defect. Quantitative analysis of labeled (or partially labeled) cells and unlabeled cells in each image revealed a significantly lower average number of cell divisions for Δ*omp19* in pancreatin-pads (*p* < 0.001 vs. *wt* in pancreatin) (Figure 4B).

Cell division requires critical regulation of the cell-cycle to coordinate genome replication and segmentation, therefore cell-cycle progression on individual bacteria was determined. While incubation of wt with pancreatin did not alter its progression along the cell-cycle, Δ*omp19* resulted in cell-cycle arrest at G1 (Figure 4C), that was evident after 3 h of incubation by the rate of cells accumulated in G1-phase (*p* < 0.001 vs. wt + pancreatin, Figure 4D). Besides, expression of cell-cycle master regulator CtrA and chaperonin GroEL were evaluated upon treatment with pancreatic elastase. Pancreatic elastase treatment increased CtrA signal in Δ*omp19*, whereas GroEL expression was similar in both strains exposed or not to proteases (Figure 4E).

Together, these results reveal that Δ*omp19* exposed to pancreatic proteases has a cell division defect that is linked to impaired progression through G1-phase and CtrA accumulation.

### Omp19 Protects Omp25 From Pancreatic Elastase Digestion

**Figure 5:**
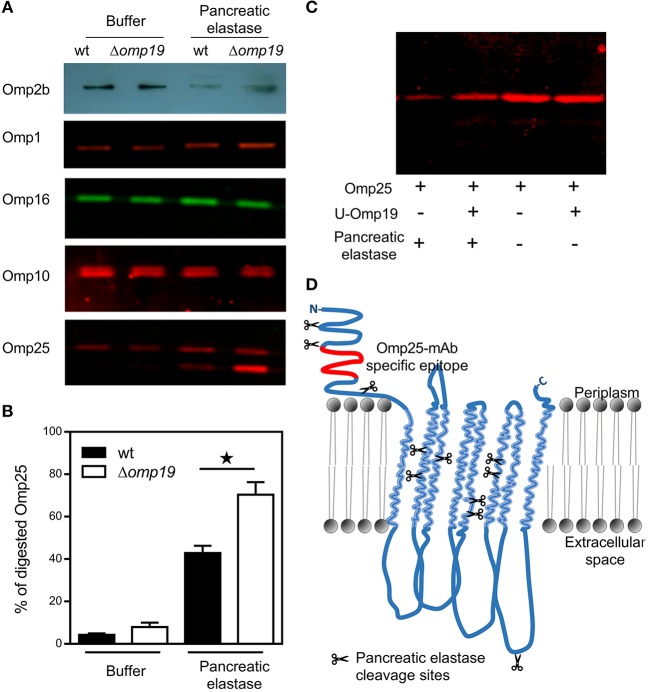
Omp19 on the *B. abortus* membrane protects Omp25 from pancreatic elastase digestion. Wt and Δ*omp19 B. abortus* strains were incubated with buffer (10 mM Tris-HCl, pH8.8) or pancreatic elastase. **(A)** Equal quantities of bacteria were subjected to SDS-page followed by western blot analysis using specific antibodies for cell envelope proteins: Omp2b, Omp1, Omp16, Omp10, and Omp25. Images are representative from two or three independent experiments. **(B)** Percentage of digested Omp25 evaluated by quantitative analysis of western blot images. Data represent pooled results from two independent experiments (^*^p < 0.05 vs. *wt B. abortus* strain in pancreatic elastase). **(C)** Recombinant purified Omp25 was incubated with pancreatic elastase with or without U-Omp19. Following incubation, each mixture of reaction was separated on SDS-PAGE followed by western blot analysis with Omp25 specific antibodies. **(D)** Graphical representation of BOCTOPUS (31) or PRED-TMBB2 (32) transmembrane β-barrel predicted topology for Omp25 with respect to the lipid bilayer representation of the *B. abortus* outer membrane. Scissors indicate predicted pancreatic elastase cleavage sites (AA or AG) on Omp25 sequence. The position of the specific epitope for the anti-Omp25 mAb used is colored in red.

As cell envelope constitutes the first contact with host-proteases, cell envelope proteins were evaluated in wt and Δ*omp19* upon protease treatment. No changes between wt and Δ*omp19* were detected upon pancreatic elastase treatment in Omp1, Omp10, or Omp16. On the contrary, in both strains Omp25 presented a lower molecular weight band and reduced Omp2b intensity upon pancreatic elastase incubation that would correspond to digested Omp25 and Omp2b, respectively (Figure 5A). While no Omp19-dependent protection of Omp2b digestion was evidenced in wt strain compared to Δ*omp19* strain, the percentage of digested Omp25 was higher in Δ*omp19* (Figure 5B), highlighting Omp19's inhibitory role of pancreatic elastase. Omp19 inhibition of pancreatic elastase digestion of Omp25 was confirmed *in vitro* using recombinant purified proteins. Pancreatic elastase digestion of rOmp25 was evidenced by a reduced Omp25-specific signal in western blot compared with the signal of rOmp25 without protease. This reduction was lower when U-Omp19 was added, indicating that U-Omp19 inhibits Omp25 digestion by pancreatic elastase (Figure 5C). Differences in the digestion pattern between Omp25 expressed on the *Brucella* membrane and recombinant Omp25, may be due to differential accessibility of pancreatic elastase cleavage sites, since in membrane associated Omp25 most cleavage sites are in predicted transmembrane regions or in loops facing the periplasm, only one cleavage site would be accessible to the protease when Omp25 is in the context of the *Brucella* membrane (Figure 5D).

These results together indicate that when Omp19 is absent, pancreatic elastase gains access to the membrane following degradation of Omp25, on the contrary under physiologic condition where Omp19 is present, *Brucella* wt can withstand this protease activity.

### Omp19 Impairs Macrophage Microsomal Proteolytic Killing of *B. abortus*

**Figure 6:**
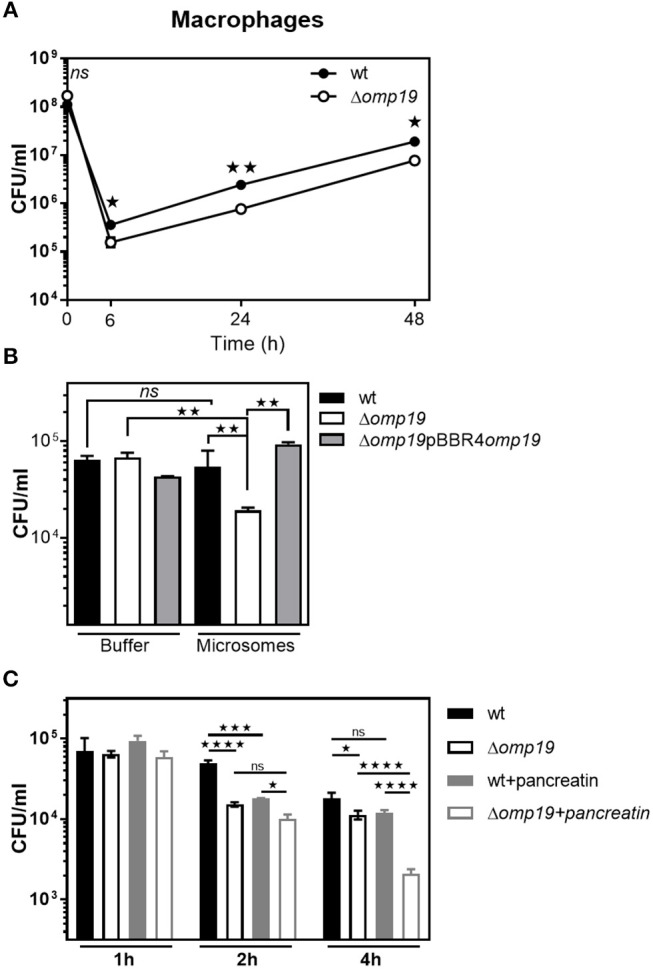
Omp19 impairs macrophage microsomal proteolytic killing of *B. abortus*. **(A)** J774 macrophages were infected (MOI 500:1) with the *B. abortus* wt or Δ*omp19* strains. At the indicated time points post-infection intracellular CFUs of *B. abortus* wt or Δ*omp19* strains were determined. (Statistical analysis was performed by two-way ANOVA followed by Bonferroni posttest to compare between the indicated groups: ^ns^*p* > 0.05; ^*^p < 0.05 or ^**^p < 0.01). **(B)** *wt*, Δ*omp19* or Δ*omp19*pBBR4*omp19 B. abortus* strains (1 × 10^5^ CFU/ml) were incubated with PBS or J774 derived microsomes at 37°C. Live bacteria (CFU/ml) were determined after 8 h by serial dilutions plating (Statistical analysis was performed by one-way ANOVA followed by Bonferroni posttest to compare between the indicated groups: ^ns^*p* > 0.05, ^**^p < 0.01). **(C)** Δ*omp19* and *wt* strains were preincubated with buffer or pancreatin for 1 h prior to infection of J774 macrophages (MOI 100:1). Intracellular CFUs were determined at 1, 2, or 4 h post-infection. (Statistical analysis was performed by two-way ANOVA followed by Bonferroni posttest to compare between the indicated groups: ^ns^*p* > 0.05; ^*^p < 0.05, ^***^p < 0.001, ^****^p < 0.0001).

Reaching the intracellular replicative niche is the next step for establishment of infection. Therefore, the ability of Δ*omp19* mutants to enter cells and replicate intracellularly was studied in professional phagocytes (Figure 6). In agreement with previous studies significant lower amounts of Δ*omp19* were found after 6, 24, and 48 h of infection in comparison to wt strain (Figure 6A). Moreover, Δ*omp19* strain was significantly more susceptible to killing by microsomal content than wt or Δ*omp19*pBBR4*omp19* (*p* < 0.01 vs. wt + microsomes or Δ*omp19*pBBR4*omp19* + microsomes) (Figure 6B), suggesting that Omp19 may protect the bacteria from lysosomal proteolysis during intracellular traffic.

When infecting through the oral route *Brucella* will reach the intracellular compartment after facing with gastrointestinal proteases, thus Δ*omp19* and *wt* strains were preincubated with pancreatin or buffer for 2 h prior to infection of J774 macrophages and intracellular bacterial counts were determined after 1, 2, or 4 h of infection. Pre-incubation with pancreatin did not affect bacterial internalization, since similar amounts of intracellular bacteria of both strains were recovered after 1 h of infection. After 4 h of infection, preincubation with pancreatin led to an increased susceptibility of Δ*omp19* to intracellular killing by macrophages, compared to pancreatin pretreated wt (*p* < 0.0001) or buffer pretreated Δ*omp19* (*p* < 0.0001) (Figure 6C). These results indicate that the sequential action of intestinal proteases followed by intracellular microsome proteolytic killing has an important effect on hampering Δ*omp19* ability to establish an intracellular niche in macrophages. Altogether these results may explain the highly attenuated phenotype of this strain when infection occurs by the oral route.

## Discussion

After consumption of infected milk or experimental oral infection, live *Brucella* are detected in fecal samples of natural host like cattle, bison, wolf and marine mammals, indicating that *Brucella* transits and pass the harsh environment of gastrointestinal tract (33).

Our results demonstrate that in mice, after oral infection (either by gavage or inoculation at the oral cavity) *Brucella* reaches the gut. After 1 h of infection brucellae were found at the lumen and epithelium of duodenum. This fast infection capacity of *Brucella* was shown in a calf ligated ileal-loop model, in which *Brucella* bacteremia was detected 30 min after intraluminal inoculation without histopathologic traces of lesions (34). *Brucella* may spread systemically from the digestive tract by transepithelial migration in mucosal epithelial barrier or through M cells (26, 34, 35).

As protease inhibitor activity against main gastrointestinal proteases was demonstrated for U-Omp19 and because of its strategic location on the outer membrane for interacting with host proteases (7–9), we speculated that Omp19 may allow *Brucella* to withstand the gastrointestinal proteolysis and infect orally. Omp19's protease inhibitor broad-specificity (8, 9) would also be advantageous regarding the different proteases that *Brucella* may encounter along infection. Like broad-spectrum serine-protease inhibitor from *Tannerella forsythia*, that may protect it from proteases from other bacteria and from the host (3).

In this work, Omp19's role in virulence in an oral infection murine model was examined. Our results showed that Omp19 expression is needed for establishment of oral acquired *B. abortus* infection. In contrast to wt, Δ*omp19* was cleared from the spleens and CLNs at 20 days post infection. Remarkably after intraperitoneal infection of mice, *omp19* deletion resulted in significant loss of virulence but the bacteria were not cleared (36, 37), this difference highlights the importance of Omp19 for *Brucella* oral infection, probably due to the huge amounts of proteases encountered when infecting through this route. Attenuation upon systemic infection and intracellularly may be due to other host-proteases action, like lysosomal proteases, to which Omp19's inhibitory activity was demonstrated (9).

Intestinal content exerted a bacteriostatic action on Δ*omp19 in vivo* and *in vitro*, revealing a protective role for Omp19 in *Brucella* against intestinal proteases. This is the first work demonstrating *in vivo* a role of a protease inhibitor in acquisition of a bacterial disease by the oral route, therefore these findings are highly relevant for foodborne infections. Interestingly, gut microbiota, that survive in this protease-rich medium, produce protease inhibitors to protect them self from exogenous proteases (38–41).

*In vitro* experiments with purified proteases shed light into the role of individual proteases in the bacteriostatic action of intestinal content. Δ*omp19*'*s* growth is hampered by the action of pancreatic elastase, indicating that inhibition of this protease by Omp19 on *B. abortus* membrane is important during the initial steps of infection. Trypsin and α-chymotrypsin have been shown to elicit antibacterial activities against *E. coli, Proteus vulgaris, Pseudomonas aeruginosa, S. aureus, Streptococcus pyogenes, and Vibrio cholerae* (42, 43), but have no effect on *B. abortus*. This resistance is Omp19-independent, indicating that it may be mediated by other mechanism.

Pancreatic proteases induce a cell division defect in Δ*omp19* that is linked to cell-cycle arrest in G1-phase. Interestingly, G1 arrest also occurs during intracellular trafficking of *B. abortus* and on starvation in *Sinorhizobium meliloti* (44, 45). Therefore, delaying initiation of DNA replication could be a common feature used by α-proteobacteria in response to harsh conditions such as infection or starvation.

In *Caulobacter crescentus*, degradation of the CtrA cell-cycle master regulator occurs at specific points in the cell-cycle. Clearance of active CtrA at the G1/S transition allows the initiation of DNA replication and cell-cycle progression (30, 46). Moreover, expression of a constitutively active stable CtrA derivative results in dominant G1 arrest (30). In *B. abortus*, the essential role of CtrA in cell division was recently confirmed (47). Thus, accumulation of CtrA in Δ*omp19* upon pancreatic protease treatment, agrees with the cell-cycle arrest in G1 induced in this strain upon treatment with proteases.

Antimicrobial functions of proteases can be due to the attack of Omps leading to loss of membrane integrity (42, 43, 48, 49). Since outer membrane proteins are exposed on the bacterial surface, they could be targets of pancreatic elastase. Among all Omps evaluated, our results indicate that Omp10, Omp16, and Omp1 of either wt or Δ*omp19* were resistant to the action of pancreatic elastase, whereas, Omp2b and Omp25 were digested by this protease. This result is consistent with protease digestion of Omps in *E. coli* or *P. aeruginosa*, in which the major Omps, OmpA, and OmpF, respectively, were degraded, while other Omps remained not affected (48, 49). Although *Brucella* Omp25 does not share identity with *E. coli* OmpA (50), topology predictions suggest that both contain similar secondary structural properties and may play a similar function (51). Notably, Omp19 expression in *Brucella* inhibited pancreatic elastase mediated Omp25 digestion. This role of Omp19 on inhibition of pancreatic elastase mediated Omp25 digestion was confirmed *in vitro* using recombinant purified proteins. Omp19 inhibition of pancreatic elastase digestion of Omp25 may explain the resistance of wt strain to the action of this protease. A similar role was described for the periplasmic protease inhibitor ecotin from *E. coli*, which reduces the bactericidal action of neutrophil elastase by protecting OmpA on the bacterial membrane from neutrophil elastase mediated digestion (2).

In this work, we found that a *Brucella omp19* deletion mutant is highly attenuated in mice after oral infection. This attenuation can be explained by bacterial increased susceptibility to host proteases met by *Brucella* during establishment of infection. Δ*omp19* has a cell division defect when exposed to pancreatic proteases that is linked to cell-cycle arrest in G1-phase, Omp25 degradation on the cell envelope and CtrA accumulation. Interestingly, a link between these three molecules was found recently, in which CtrA can bind the promotor of *omp25* and *omp19*. The same work demonstrates that CtrA controls the expression of Omp25 (47), therefore the increment in Omp25 intensity in Δ*omp19* upon pancreatic elastase treatment may be explained by the increment in CtrA expression.

Upon entry into mammalian cells, the intracellular pathogen *Brucella abortus* resides within a membrane-bound compartment, the *Brucella*-containing vacuole (BCV), the maturation of which is controlled by the bacterium to generate a replicative organelle derived from the endoplasmic reticulum (ER). BCVs traffic along the endocytic pathway and fuse with lysosomes, and such fusion events are required for further maturation of BCVs into an ER-derived replicative organelle (52). Thus, the role of Omp19 for intracellular survival was studied. In agreement with previous work (36, 37), Δ*omp19* was attenuated inside macrophages. This attenuation may be due to increased susceptibility to intracellular proteases when lacking Omp19. This hypothesis is reinforced by the fact that Omp19 is able to inhibit lysosomal proteases (9) and here we demonstrated that Δ*omp19* is more susceptible to proteolytic killing by microsomes from macrophages. This increased susceptibility may explain the slight attenuation for systemic infections in mice, in which high persistence of Δ*omp19* was shown after 4 weeks of infection (36, 37). An additive effect in increasing susceptibility of Δ*omp19* was observed when the strains were preincubated with pancreatic proteases prior to infection of macrophages. This increased susceptibility may account for the high attenuation of Δ*omp19* after *in vivo* oral infection. Therefore, Omp19 would allow *Brucella* spp. to bypass lysosomal destruction thus enabling *Brucella* to survive inside macrophages and start a chronic infection.

Overall, this study demonstrates that the protease inhibitor Omp19 confers *B. abortus* the ability to resist the action of proteases. Together with urease that may protect *Brucella* from stomach low pH (17) and cholylglycine hydrolase that confers resistance to bile salts (18), Omp19 by inhibiting intestinal and intracellular proteases contributes to the establishment of chronic infection through the oral route.

## Contribution to the Field Statement

Understanding how infectious pathogens spread is critical to prevent infectious diseases. One of the principal ways in which human and animal Brucellosis is acquired, is the oral route. This implies that Brucellae must survive the harsh conditions along the gastrointestinal tract before reaching the mononuclear phagocytes to form a replicative niche. In this work, we demonstrate that *Brucella* has a lipoprotein, called Omp19, which is a protease inhibitor, that enables it to survive the proteolytical action of gut digestive and microsomal derived proteases. The significance of our research is in identifying a new mechanism involved in virulence in oral acquired Brucellosis, that will enhance our understanding of *Brucella* pathogenesis and would serve as a model for other food-borne diseases.

## Data Availability

The raw data supporting the conclusions of this manuscript will be made available by the authors, without undue reservation, to any qualified researcher.

## Ethics Statement

Protocols of this study agreed with international ethical standards for animal experimentation (Helsinki Declaration and amendments, Amsterdam Protocol of welfare and animal protection and NIH guidelines for the Care and Use of Laboratory Animals). Protocols of this study were approved by the Institutional Committee for the Care and Use of Experimentation Animals from UNSAM (CICUAE-UNSAM_N°04/2014).

## Author Contributions

KP, MC, and JC designed the experiments. Funding acquisition was done by JC. MC performed most laboratory assays with assistance from KP, FG, LB, DR, and LC. MR performed susceptibility to bile salts assays and some of the J774 macrophage infection assays. KP, JC, and MC performed all statistical analysis. DC provided bacterial strains, materials and, together with JC and KP contributed with their expertise on the subject. KP, MC, and JC interpreted all results. KP and JC wrote the manuscript. All authors reviewed, commented, and approved the manuscript.

### Conflict of Interest Statement

The authors declare that the research was conducted in the absence of any commercial or financial relationships that could be construed as a potential conflict of interest.
