# Supplementary material for: Omp19 Enables Brucella abortus to Evade the Antimicrobial Activity From Host's Proteolytic Defense System
Source: Front Immunol. 2019 Jun 26;10:1436. doi: 10.3389/fimmu.2019.01436 (PMC6607954; doi:10.3389/fimmu.2019.01436)
Supplement: Supplementary file 1 [file Data_Sheet_1.pdf]

Supporting Information
S1 Fig

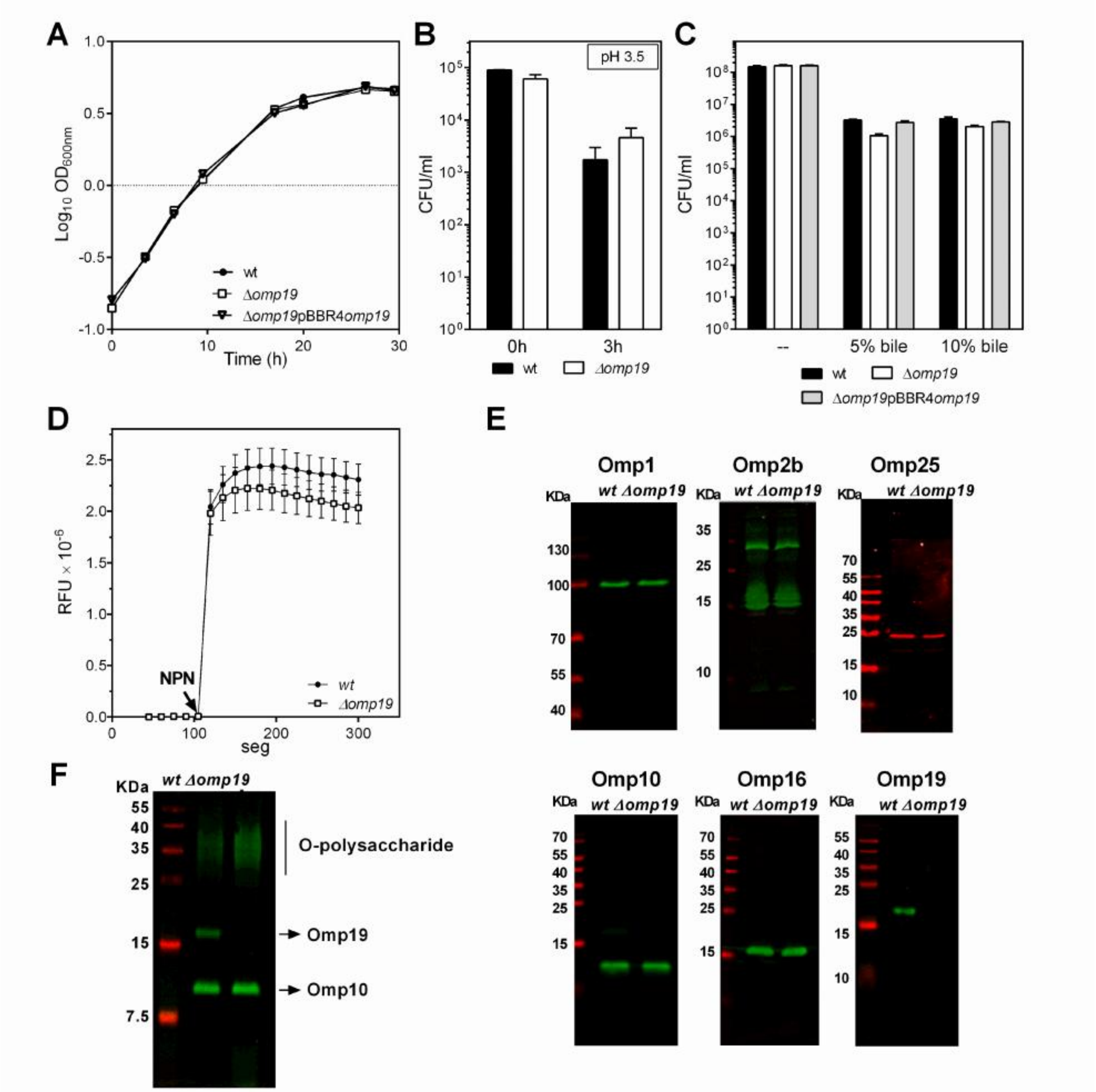

S1 Fig. Growth and membrane properties of *B. abortus* WT and mutant strains. (A) Growth curves of wt, Δ*omp19* and Δ*omp19*pBBR4*omp19* *B. abortus* cultured in TSB at 37ºC. The Bacterial growth was monitored by measuring the optical density at 600 nm. **(B)** Susceptibility of wt and Δ*omp19* *B. abortus* to low pH. Bacteria were incubated in buffer at pH 3.5 at 37 °C. Data indicate the number of viable bacteria after 3 h in triplicate experiments. **(C)** Growth of wt, Δ*omp19* and Δ*omp19*-omp19 *B. abortus* in TSB alone or TSB containing different concentrations of bovine bile (5% or 10%) at 37°C. Data indicate the number of viable bacteria after 18 h of culture in each medium in triplicate experiments. **(D)** Measurement of outer membrane permeability of wt and Δ*omp19* *B. abortus* with N-phenyl-naphthylamine (NPN) uptake assay. 3 × 10^9 CFU/ml of wt and Δ*omp19* *B. abortus* strains were suspended in PBS and 10 µM of NPN was added. The fluorescence generated was measured at an excitation wavelength of 350 nm and an emission wavelength of 420 nm. The arrow indicates the time of addition of NPN (➝). RFU: relative fluorescence units. Western blot analysis of **(E)** outer membrane

proteins (Omp1, Omp2b, Omp25, Omp10, Omp16 and Omp19) and **(F)** lipopolysaccharide O-antigen in wt and Δ*omp19 B. abortus* strains. Bacteria were grown in TSB and harvested in stationary phase. Equivalent bacterial pellets were suspended in Laemmli buffer and samples were subjected to SDS-PAGE. Proteins were transferred onto nitrocellulose membranes using a semi-dry transfer procedure. Immunoblotting was performed using mouse monoclonal antibodies against Omp1, Omp2b, Omp25, Omp10, Omp16 and Omp19 (kindly provided by Dr. Axel Cloeckaert) and mouse anti-O-polysaccharide monoclonal antibody M84 (kindly provided by Dr. K Nielsen).
